# Special Issue “Rumen Microbial Communities”: Editorial

**DOI:** 10.3390/microorganisms11040919

**Published:** 2023-04-01

**Authors:** Benoit St-Pierre

**Affiliations:** Department of Animal Science, South Dakota State University, Animal Science Complex, Box 2170, Brookings, SD 57007, USA; benoit.st-pierre@sdstate.edu

Ruminants represent a highly successful group of herbivores that have not only evolved to thrive across a wide range of habitats, but have also played a central role throughout human history [1]. Even to this day, the ability of domesticated ruminants to transform inedible plant biomass into products that can be consumed or utilized by humans is essential in meeting the demands for animal protein by a rapidly growing and urbanizing global population [2]. Ruminants are able to metabolize cellulosic biomass because of the metabolic activities of symbiotic microbial communities that reside in the rumen compartment of their gastrointestinal tract [3,4]. Elucidating the complexities of ruminal microbial communities remains a challenging subject for basic and applied scientists. The Special Issue ‘Rumen Microbial Communities’ featured ten peer-reviewed research articles that contributed to expanding our knowledge of rumen symbionts in the context of ruminant production, adaptation to natural habitats as well as basic physiology.

Ruminant livestock production is an integral part of the agriculture sector. Since microbial metabolism precedes host digestion in ruminants [4], a great deal of effort has been dedicated to further elucidating the development of the rumen environment in young animals; improvements in reducing the weaning period or modulating the development of the rumen microbial ecosystem would for instance greatly benefit ruminant livestock production. In this context, Amat et al. (2021) evaluated whether the ruminal microbiota, as well as nasopharyngeal and vaginal bacterial communities, would be affected in virgin heifers that were raised from dams that were restricted in maternal gain during the first trimester of gestation [5]. Wang et al. (2022) investigated the temporal dynamics of rumen microbiota in early weaned lambs, as this area remains poorly explored compared to our knowledge on conventionally weaned lambs [6].

In a continued effort to increase the efficiency of ruminant production, elucidating the response of rumen microbial communities to transitions in diet or to supplementation with particular nutrients or direct-fed microbials remains of high interest in this field, as demonstrated by the four articles on this topic. In their study, Hao et al. (2021) aimed to gain a deeper understanding of the effects of diet and age on rumen function and on the ruminal bacteria communities of dairy cattle [7]. On their part, Cancino-Padilla et al. (2021) investigated the long-term effects of supplementing ruminant diets with olive oil on the rumen microbiome of dairy cows [8]. While supplementation with dietary lipids can be for the purpose of increasing the energy density in a diet, it can also be used as a strategy to increase the beneficial fatty acid profile in ruminant products. In this context, Mavrommatis et al. (2021) investigated the effects of supplementation with the PUFA-rich marine microalgae *Schizochytrium* spp. on the goat rumen microbiome [9]. Finally, the report by Zhang et al. (2021) described a study on the effects of thiamine supplementation on the abundance of the microorganisms and enzymes involved in carbohydrate degradation in the rumen of goats [10].

Gaining deeper insights on wild ruminants is also a critical line of research in this field. This information can contribute to the development of strategies for species and habitat conservation, such as the report by Park et al. (2021) on populations of the Long-Tailed Goral (*Naemorhedus caudatus*), an endangered species found in the mountains of eastern and northern Asia. In this study, seven populations of Long-Tailed Goral from South Korea were assigned to three groups based on the composition of their gut microbiome [11]. Another important contribution from investigating wild ruminants is the potential for insights on basic ruminal functions that not only contribute to our fundamental knowledge base, but also can benefit production from traditional domesticated ruminant species or can be used for biotechnological applications such as conversion of plant biomass into biofuels. In this context, Wu et al. (2022) explored the metabolic potential of ruminal microorganisms in the Muskox (*Ovibos moschatus*). As the largest herbivore in the High Arctic, it is an example of a ruminant that has evolved to efficiently utilize the scarce and highly lignified forages in its habitat; it was thus hypothesized to harbor previously uncharacterized microbial enzymes that would be capable of efficiently metabolizing plant biomass [12].

Finally, the other articles in this Special Issue aimed at providing insights on more basic or fundamental aspects of the rumen microbial environment. Pacifico et al. (2021) performed a meta-analysis using 11 publicly available datasets from studies on rumen epimural microbiota [13]. These sub-communities play a number of important roles in the rumen, but as they consist of microorganisms that are firmly attached to the rumen wall, it is very challenging to investigate them. On their part, Bandarupalli and St-Pierre (2020) described the identification of a previously uncharacterized candidate strain of *Prevotella albensis*, which was identified based on its ability to grow from rumen fluid batch cultures with starch as the only substrate provided; this metabolic capacity was confirmed by a metagenomics assessment of its encoded proteome [14].

## References

[1] Hackmann T.J., Spain J.N. (2010). Invited Review: Ruminant ecology and evolution: Perspectives useful to ruminant livestock research and production. J. Dairy Sci..

[2] Thornton P.K. (2010). Livestock production: Recent trends, future prospects. Philos. Trans. R. Soc. Lond. B Biol. Sci..

[3] Hofmann R.R. (1989). Evolutionary steps of ecophysiological adaptation and diversification of ruminants: A comparative view of their digestive system. Oecologia.

[4] Macfarlane G.T., Macfarlane S. (1993). Factors affecting fermentation reactions in the large bowel. Proc. Nutr. Soc..

[5] Amat S., Holman D.B., Schmidt K., Menezes A.C.B., Baumgaertner F., Winders T., Kirsch J.D., Liu T., Schwinghamer T.D., Sedivec K.K. (2021). The Nasopharyngeal, Ruminal, and Vaginal Microbiota and the Core Taxa Shared across These Microbiomes in Virgin Yearling Heifers Exposed to Divergent in Utero Nutrition during Their First Trimester of Gestation and in Pregnant Beef Heifers in Response to Mineral Supplementation. Microorganisms.

[6] Wang S., Chai J., Zhao G., Zhang N., Cui K., Bi Y., Ma T., Tu Y., Diao Q. (2022). The Temporal Dynamics of Rumen Microbiota in Early Weaned Lambs. Microorganisms.

[7] Hao Y., Gong Y., Huang S., Ji S., Wang W., Wang Y., Yang H., Cao Z., Li S. (2021). Effects of Age, Diet CP, NDF, EE, and Starch on the Rumen Bacteria Community and Function in Dairy Cattle. Microorganisms.

[8] Cancino-Padilla N., Catalán N., Siu-Ting K., Creevey C.J., Huws S.A., Romero J., Vargas-Bello-Pérez E. (2021). Long-Term Effects of Dietary Supplementation with Olive Oil and Hydrogenated Vegetable Oil on the Rumen Microbiome of Dairy Cows. Microorganisms.

[9] Mavrommatis A., Skliros D., Flemetakis E., Tsiplakou E. (2021). Changes in the Rumen Bacteriome Structure and Enzymatic Activities of Goats in Response to Dietary Supplementation with *Schizochytrium* spp.. Microorganisms.

[10] Zhang Y., Wang C., Peng A., Zhang H., Wang H. (2021). Metagenomic Insight: Dietary Thiamine Supplementation Promoted the Growth of Carbohydrate-Associated Microorganisms and Enzymes in the Rumen of Saanen Goats Fed High-Concentrate Diets. Microorganisms.

[11] Park C.E., Cho B.-J., Kim M.-J., Park H.C., Shin J.-H. (2021). Geographical Relationships between Long-Tailed Goral (*Naemorhedus caudatus*) Populations Based on Gut Microbiome Analysis. Microorganisms.

[12] Wu X., Elekwachi C.O., Bai S., Luo Y., Zhang K., Forster R.J. (2022). Characterizing the Alteration in Rumen Microbiome and Carbohydrate-Active Enzymes Profile with Forage of Muskoxen Rumen through Comparative Metatranscriptomics. Microorganisms.

[13] Pacífico C., Petri R.M., Ricci S., Mickdam E., Wetzels S.U., Neubauer V., Zebeli Q. (2021). Unveiling the Bovine Epimural Microbiota Composition and Putative Function. Microorganisms.

[14] Bandarupalli V.V.K., St-Pierre B. (2020). Identification of a candidate starch utilizing strain of *Prevotella albensis* from bovine rumen. Microorganisms.

